# Colonic Explosion During Argon Plasma Coagulation: A Systematic Review of Risk Factors, Prevention, and Management

**DOI:** 10.1002/jgh3.70413

**Published:** 2026-10-30

**Authors:** Fariha Hasan, Ayesha Liaquat, Ishan Bhalgat, Ahmer Khan, Hareesha Rishab Bharadwaj, Dushyant Singh Dahiya, Shahryar Khan, Christina Tofani, Apeksha Shah, Anthony Infantolino

**Affiliations:** ^1^ Department of Internal Medicine Cooper University Hospital Camden New Jersey USA; ^2^ Department of Internal Medicine Dow University of Health Sciences Karachi Pakistan; ^3^ Cooper Medical School of Rowan University Camden New Jersey USA; ^4^ Department of Gastroenterology Jefferson Einstein Hospital Philadelphia Pennsylvania USA; ^5^ Faculty of Biology, Medicine and Health University of Manchester Manchester UK; ^6^ Division of Gastroenterology, Hepatology and Nutrition University of Kansas School of Medicine Kansas City Kansas USA; ^7^ Department of Internal Medicine University of Kansas Health System Kansas City Kansas USA; ^8^ Department of Gastroenterology Cooper University Hospital Camden New Jersey USA

## Abstract

**Introduction:**

Argon plasma coagulation (APC) is a non‐contact electrocoagulation technique widely used for hemostasis in gastrointestinal endoscopy. Despite its safety, rare but catastrophic complications such as colonic explosions have been reported. This systematic review aimed to synthesize available evidence on APC‐associated colonic explosions, identify risk factors, and highlight preventive and management strategies.

**Methods:**

Following PRISMA guidelines, PubMed, Embase, Web of Science, and Cochrane CENTRAL were searched through August 2025 for studies reporting colonic explosions during APC. Eligible studies included clinical reports describing APC use in gastrointestinal procedures with outcomes of explosion or perforation. Data were extracted on patient demographics, procedural context, bowel preparation, and clinical outcomes.

**Results:**

Ten studies, comprising 12 cases, were included. Explosions most frequently occurred during APC for radiation proctitis, adenoma, or angiodysplasia, predominantly after inadequate bowel preparation or use of enemas alone. Combustible gases—chiefly hydrogen and methane—were identified as the principal mediators. Perforation occurred in half of the cases, all of which were surgically repaired, with no reported fatalities.

**Conclusion:**

Although rare, colonic explosion during APC remains a preventable event. Complete bowel cleansing with oral polyethylene glycol‐based agents, avoidance of air insufflation, and adherence to safety protocols are essential to minimize risk. Standardized preparation guidelines and further research are warranted.

## 1 Introduction

Argon plasma coagulation (APC) is an endoscopic non‐contact thermal coagulative technique in which chemically inert argon gas is fed to an electrode, ionized, and delivered in the form of a current to the target tissue. The inherent resistance of human tissue (i.e., < 300 Ohms in the colon) causes the consequent electrical heating, cauterization, and ablation of tissue by the process of ohmic heating [1]. Accordingly, in gastrointestinal settings, APC is often used to control a variety of bleeding syndromes, such as angiodysplasia, bleeding ulcers, gastric antral vascular ectasia, or radiation proctitis [2].

APC is widely regarded as a safe and user‐friendly procedure during endoscopy, offering the advantage of a controlled coagulation depth of a few millimeters. Additionally, as a non‐contact modality, APC is well‐suited to treat large or uneven surface areas, such as the colon or neoplastic tissue. Nonetheless, despite a favorable safety profile, complications such as fistulation, bowel perforation, and, more notably, colonic explosion have been reported in the literature [3]. While not exclusive to APC (i.e., electrocautery or polypectomy), the ohmic tissue heating associated with APC serves as a source of ignition when accumulated combustible gases (e.g., hydrogen, methane, and oxygen) in the bowel, resulting in colonic explosion with or without perforation. Inadequate local bowel preparation can result in the accumulation of combustible gases within the colon, a phenomenon reported with both fermentable and non‐fermentable preparatory agents. Residual stool following insufficient preparation can serve as a substrate for bacterial fermentation, generating additional combustible gases. Furthermore, the use of non‐ or partially‐absorbable osmotic laxatives, such as mannitol or sorbitol, may promote the fermentation processes that produce these gases [4]. Lastly, accidental air insufflation or chemical reactions during enema administration may further contribute to intraluminal gas presence, while the liberation of dissolved gases can occur as a result of laxative administration.

Historically, reports of colonic explosion during APC have been isolated to individual case reports, resulting in a literature gap of comprehensive synthesis and contextual understanding of these events. Accordingly, clear identification of risk factors and the development of best practice guidelines for the prevention, mitigation, and management of APC‐associated colonic explosion are lacking. To address this literature gap, the present systematic review aims to synthesize available evidence of colonic explosion and perforation associated with the use of APC in gastrointestinal procedures, highlighting associated risk factors, prevention strategies, and mitigation approaches.

## 2 Methods

This systematic review was conducted in accordance with the Cochrane Handbook for Systematic Reviews of Interventions and adhered to the Preferred Reporting Items for Systematic Reviews and Meta‐Analyses (PRISMA) guidelines [5, 6]. This systematic review was not prospectively registered in a database.

### 2.1 Data Sources and Search Strategy

An extensive literature search was conducted using electronic databases such as PubMed, Embase, Web of Science, and Cochrane CENTRAL from database inception till August 2025. The following keywords were used as a search string: “Argon Plasma Coagulation,” “colonic explosion,” “colonoscopy,” “colon,” “adenoma,” “polyp,” and “radiation proctitis.” We also manually screened the reference lists of included studies and relevant systematic reviews. The final search strategy is given in Table S1.

### 2.2 Eligibility Criteria

The primary outcome was colonic explosion during APC, while the secondary outcome included bowel perforation. For a study to be included in this systematic review, the following criteria were considered necessary: discussed the use of APC in gastrointestinal procedures, reported incidents or case studies of colonic explosions during APC, and provided data on prevention strategies and safety protocols. Studies were excluded if they focused on non‐gastrointestinal applications of APC or did not provide data on colonic explosions or associated risk factors.

### 2.3 Study Selection and Data Extraction

Two independent reviewers (L.A. and I.B.) selected studies and extracted data; any discrepancies were resolved by a third reviewer (H.F.). The initial search yielded 108 records, of which 14 studies were assessed for eligibility after screening. Among these, 4 studies were excluded at the full‐text stage as they did not investigate colonic explosion and therefore did not meet the predefined outcome inclusion criteria. The schematic diagram of study selection is illustrated in Figure 1. The included studies comprised eight case reports, one case series, and one prospective cohort study conducted across diverse geographical regions, including France, Germany, United Kingdom, Greece, and India. Data were extracted using a standardized form that captured information on study characteristics (e.g., authors, year of publication, study design, region), patient characteristics (e.g., age, sex, type of procedure), and primary and secondary outcomes.

**FIGURE 1:**
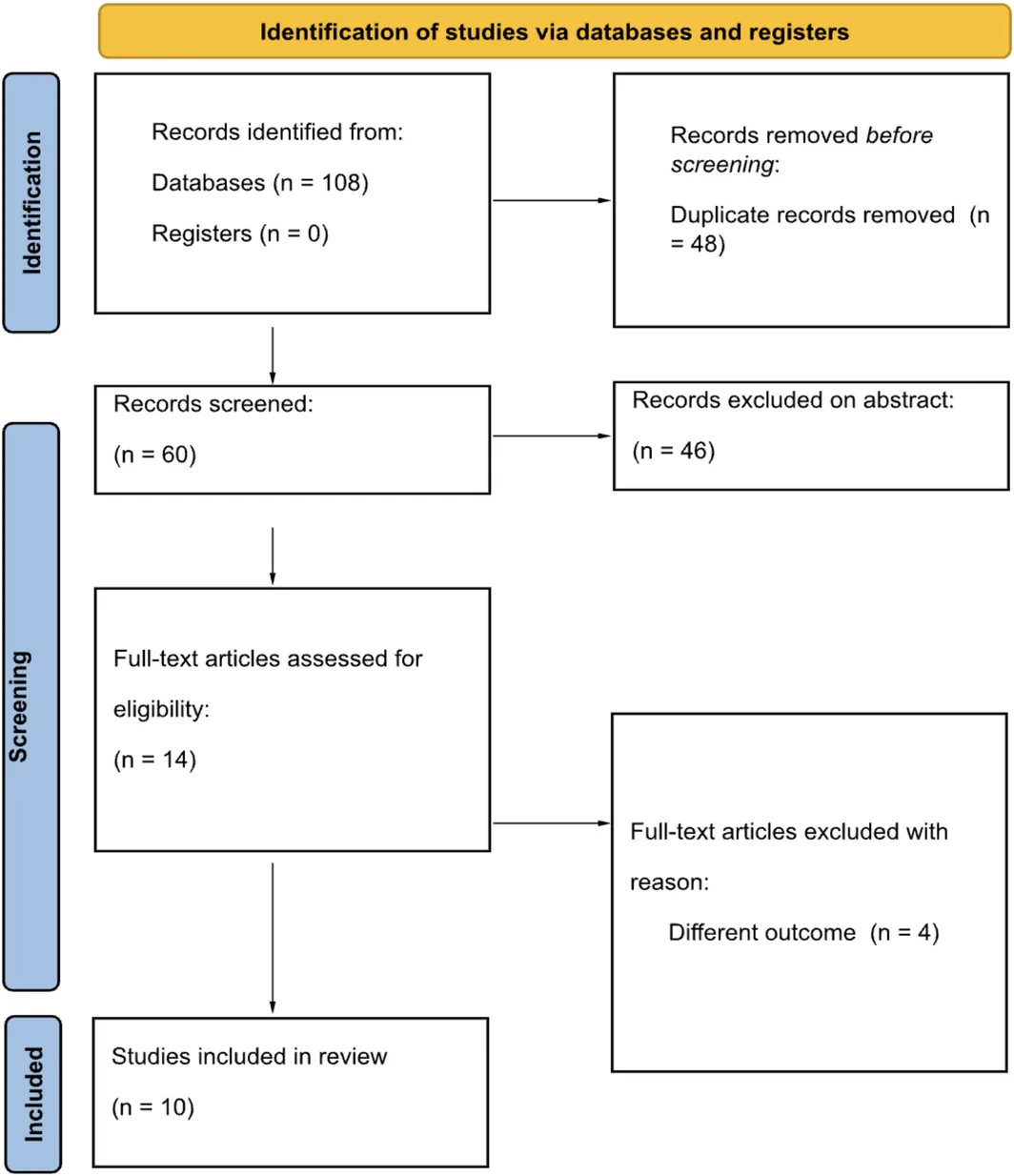
PRISMA flow chart.

### 2.4 Risk‐of‐Bias and Quality Assessment

The risk of bias in the included cohort study was assessed using the Newcastle‐Ottawa Scale, as shown in Table S2. This scale evaluates studies based on eight criteria across three domains: selection of study groups, comparability, and ascertainment of exposure or outcome. The quality assessment of the included case series was done using the Joanna Briggs Institute (JBI) critical appraisal tool, which includes 10 questions addressing the internal validity and risk of bias of case series designs, as shown in Table S3.

## 3 Results

### 3.1 Mechanism

“Explosion” as defined by Manner et al. [7] is a sudden release of energy with a subsequent increase in temperature and pressure. A limiting concentration of oxygen (LOC) and an ignition source can cause an explosion in a potentially explosive environment with combustible gases such as hydrogen and methane [8]. However, for combustible gases to actually cause an explosion, their concentration must be within the lower (LEL) and upper explosion levels (UEL). Hydrogen has an LEL, UEL, and LOC of 4%, 75%, and 4%, respectively, while for methane, these values are 4.4%, 16.3%, and 10.7%, respectively. The explosion area of hydrogen is significantly greater than that of methane, making the likelihood of an explosion with hydrogen higher, as depicted in Figure 2. However, since the enthalpy of combustion of methane (Δ*H* = −802 kJ/mol) is more than that of hydrogen (Δ*H* = −572.05 kJ/mol), it is more damaging [7].

**FIGURE 2:**
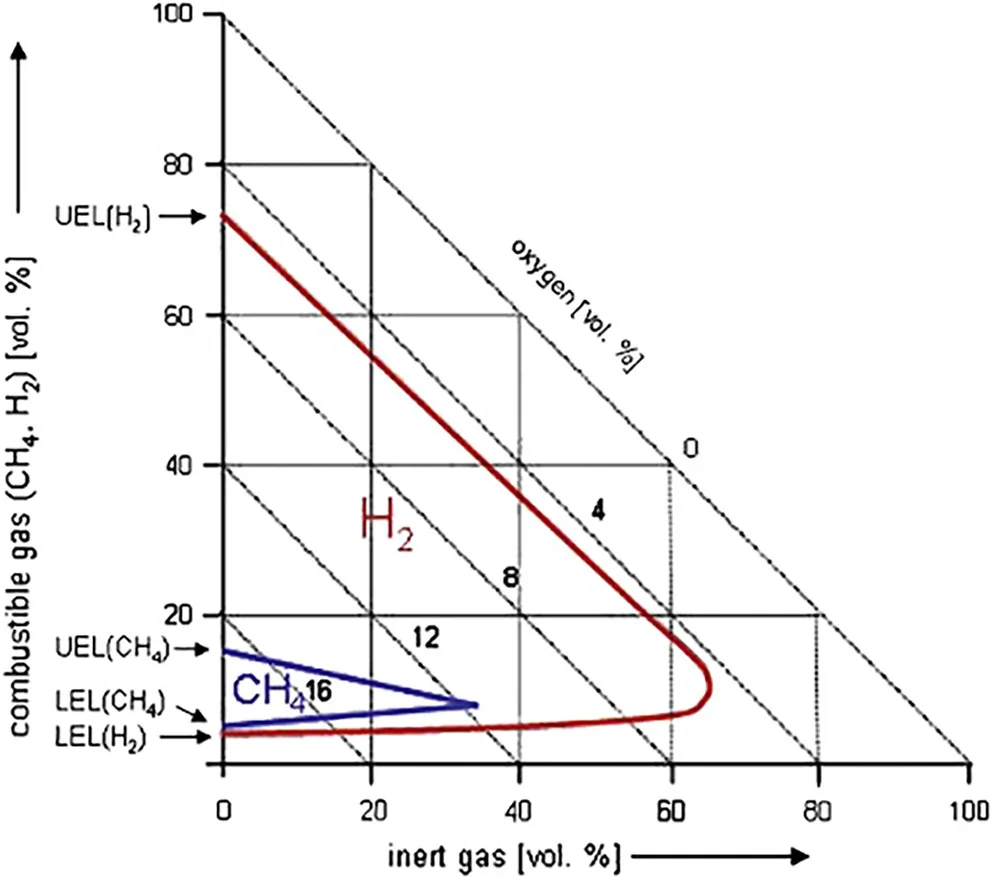
A graphical depiction of the explosive areas in a ternary system comprising a combustible gas [CH_4_ (methane) or H_2_ (hydrogen)], oxygen, and inert gas [9].

In the gastrointestinal tract, only the colon has been found to have comparable concentrations of these gases, which are produced from the fermentation of non‐absorbable (e.g., mannitol) or partially absorbable (e.g., sorbitol) carbohydrates by gut bacteria [7]. APC provides non‐contact anticoagulation by delivering high‐frequency current via ionized argon gas [10]. This creates a thermal effect in target issues and can act as an ignition source in such a potentially explosive environment, leading to an explosion.

### 3.2 Study Details

A total of 12 cases of colonic explosion during APC were reported, as given in Table 1 [9, 11, 12, 13, 14, 15, 16, 17, 18, 19]. In these cases, APC was used therapeutically for radiation proctitis, stricture, adenoma, adenocarcinoma, angiodysplasia, and rectal ulcer. The incidence of colonic explosion was significantly higher in patients with a locally prepared colon as compared to oral preparations. Most of these cases occurred in the context of inadequate bowel preparation, and the primary risk factor identified was the subsequent high concentrations of combustible gases like hydrogen and methane. In 10 out of 12 cases, an enema without any fermentable agent was used for colon preparation with no full bowel preparation. Complications associated with colonic explosion included perforation in six cases, all of which were repaired with surgery; sigmoid laceration in one case, which was repaired with Hartmann's intervention; and extensive superficially bleeding rectal and sigmoid ulcers in another case, managed with IV fluids and tramadol. No deaths following colonic explosion were reported in any of the cases.

**TABLE 1:** Overview of cases of colonic explosion during treatment with APC.

Study, year	Region	Type of study	Cases of explosion	Patient characteristics	Procedure	Diagnosis	Bowel preparation agent	Complications
Zinsser, 1999	Germany	Case report	1	85‐year‐old woman	Sigmoidoscopy	Malignant stricture of the rectosigmoid, with remaining size of lumen 8 mm	Sodium phosphate enemas	Explosion without perforation
Sousann, 2003	France	Case report	1	68‐year‐old woman	Sigmoidoscopy	Hemorrhagic radiation‐induced proctitis with chronic hemorrhage	Sterculia enemas	Explosion with perforation at rectosigmoid junction; repaired with surgery
Pichon, 2004	France	Case report	1	82‐year‐old woman	Colonoscopy	Tubuloovillous adenoma of the sigmoid colon	Saline enemas	3 sigmoid perforations; repaired with celioscopic suturing
Ben‐Sousann, 2004	France	Case series	3	NR	Sigmoidoscopy	Radiation proctitis	Enema	Patient 1: 2 successive explosions; Patient 2: Explosion with perforation, treated with surgery
Townshend, 2007	United Kingdom	Case report	1	95‐year‐old woman	Sigmoidoscopy	Rectal villous adenoma with bleeding	Sodium phosphate enema	2 perforations in the sigmoid colon; repaired with Hartmann's procedure
Nurnberg, 2007	Germany	Case report	1	69‐year‐old patient	Colonoscopy	Angiodysplasias in the ascending colon	Macrogol solution	2 perforations of ascending colon and cecum; repaired with right‐sided hemicolectomy
Seth, 2009	India	Case report	1	42‐year‐old woman	Sigmoidoscopy	Radiation proctitis	Oral saline laxative containing monosodium and disodium phosphate	Explosion without perforation. Extensive superficially bleeding ulcers in rectum and sigmoid colon; managed with IV fluids and tramadol
Karamanolis, 2009	Greece	Prospective Cohort	1	NR	NR	Radiation proctitis	Enema	Explosion without perforation
Trastulli, 2012	Italy	Case report	1	73‐year‐old patient	NR	Rectal angiodysplasia and bleeding arising following radiotherapy performed for prostate cancer	Evacuative micro‐enema without the addition of oral preparations	Explosion of sigma with perforation; repaired with Hartmann intervention
Shiha, 2021	United Kingdom	Case report	1	72‐year‐old man	Sigmoidoscopy	Rectal ulcer and two areas of actively bleeding angiodysplasia	Phosphate enema	Explosion without perforation; managed with antibiotics, parenteral nutrition, and granulocyte‐colony stimulating factor

## 4 Discussion

This review identified 10 key studies reporting 12 cases of colonic explosion during APC, offering valuable insights into the occurrence and prevention of this rare but catastrophic complication, which warrants careful attention during APC procedures. In 1954, the first case of colonic explosion during electrodesiccation was reported by Levy [20]. Subsequently, several reports of colonic explosions during APC have been published (Table 1). Higher‐than‐normal combustible gas concentrations are considered the initiating factor for such explosions. Mannitol, a fermentable colon preparation agent that degrades to produce hydrogen and methane, was once the mainstay of colon preparation but is no longer used due to published cases of colon explosions associated with its use [21, 22]. However, the findings of our systematic review show that inadequate bowel preparation, most commonly with enemas that notably did not contain any fermentable agents, is the main cause of colonic explosion. An unprepared or inadequately prepared bowel leads to residual stool, which can have up to 30% methane and 44% hydrogen [23]. In the case reported by Seth et al., a colonic explosion with superficial bleeding rectal ulcers was observed in a 42‐year‐old woman with radiation proctitis. The colon was prepared with an oral saline laxative containing monosodium and disodium phosphate, but liquid stool was observed in the rectum during colonoscopy. While there is evidence supporting the effectiveness of oral bowel preparation in reducing combustible gas concentrations in the colon [24, 25], this case highlights the importance of thorough bowel cleansing before APC regardless of the chemical cleansing agent used [16], an observation echoed by Ben‐Soussan et al. and Nurnberg et al. where the latter reported the first case of colonic explosion in a patient prepared with oral macrogol solution [13, 15]. Therefore, it is essential to ensure complete bowel preparation of the colon before every APC procedure and strictly monitor combustible gas concentrations throughout the process.

Additionally, the European Society of Gastrointestinal Endoscopy (ESGE) guidelines recommend insufflation of an inert gas such as carbon dioxide instead of air after complete bowel cleansing, as the presence of oxygen is a prerequisite for explosion. This recommendation has been shown to reduce but not eliminate the risk of colonic explosion [19, 26, 27]. A potential alternative strategy to minimize the concentrations of combustible gases in the colon is to prevent the bacterial fermentation of intestinal nutrients by administering antibiotics before endoscopic procedures. This approach remains debatable, as there is insufficient evidence supporting a lowered risk of colonic explosion with antibiotic use [16]. Pre‐procedure mitigation strategies, such as an appropriate diet to reduce meteorism and using aides such as a rectal tube to remove gas pockets in the colon, especially in the setting of stenosis, may also help curb the risk of colonic explosion [18]. Despite all these precautions, given the unpredictable nature and volume of the intracolonic gases, it is not possible to obviate the risk of this complication during APC. Therefore, this risk should be communicated and included in the informed consent [7, 16].

While our review compiles the latest data from a variety of study designs and offers a comprehensive overview of risk factors of colonic explosion during APC and the prevention strategies, it is limited by the heterogeneity of the included study designs and the rarity of the outcome reported. These factors may restrict the generalizability of the findings of our review.

Nonetheless, the findings of our review underscore the need for stringent bowel preparation prior to APC procedures and strict monitoring of combustible gas concentrations throughout the process. Given the catastrophic and mostly preventable nature of this complication, improved education and training of physicians is paramount. In addition to ensuring complete bowel preparation, physicians should be encouraged to use oral bowel preparations, such as polyethylene glycol (PEG)‐based agents, which have been shown to lower the hydrogen and methane concentrations, unlike the use of an enema alone. This is particularly important for repeat procedures, which is when most of the reported explosions with APC therapy occurred [28, 29].

## 5 Conclusion

APC is a valuable tool in endoscopy with expanding uses, but the rare yet dreaded risk of colonic explosion warrants careful attention and necessitates strict adherence to safety protocols. To mitigate this risk, physicians should be educated and trained to prioritize and perform proper bowel cleansing with oral preparation agents, monitor combustible gas concentrations, and avoid high oxygen environments during APC. Currently, there is no consensus on the standard practice for bowel preparation before APC. Therefore, more studies are required to establish standardized guidelines for bowel preparation with appropriate cleansing agents. Lastly, further research is needed to explore and develop alternative cautery techniques that are efficacious, safer, and have a lower risk of complications.

## Funding

The authors have nothing to report.

## Conflicts of Interest

The authors declare no conflicts of interest.

## Supporting information


**Table S1:** Search strategy.
**Table S2:** Quality assessment of the included prospective Cohort studies using the NewCastle Ottawa Scale (NOS).
**Table S3:** Results of Joanna Briggs Institute (JBI) critical appraisal tool for the quality assessment of the included case series.

## Data Availability

The data that support the findings of this study are available from the corresponding author upon reasonable request.
