# Supplementary material for: Colonic Explosion During Argon Plasma Coagulation: A Systematic Review of Risk Factors, Prevention, and Management
Source: JGH Open. 2026 Oct 30;10(11):e70413. doi: 10.1002/jgh3.70413 (PMC13722129; doi:10.1002/jgh3.70413)
Supplement: Supplementary file 1 [file JGH3-10-e70413-s001.docx]

Supplemental Table 1: **Search Strategy**

| **Database** | **Search Strategy/Keywords** | **Articles Retrieved** |
| --- | --- | --- |
| PubMed | (Explosion OR Colonic explosion) AND (Colonoscopy OR Colon OR Adenoma OR Polyp OR Radiation Proctitis OR Gastrointestinal Bleeding OR Tumor OR Stenosis) AND (Argon Plasma Coagulation OR Argon Cautery OR Electrocautery OR Cautery OR Cauterization) | 44 |
| Embase | (Explosion OR Colonic explosion) AND (Colonoscopy OR Colon OR Adenoma OR Polyp OR Radiation Proctitis OR Gastrointestinal Bleeding OR Tumor OR Stenosis) AND (Argon Plasma Coagulation OR Argon Cautery OR Electrocautery OR Cautery OR Cauterization) | 44 |
| Web of Science | (Explosion OR Colonic explosion) AND (Colonoscopy OR Colon OR Adenoma OR Polyp OR Radiation Proctitis OR Gastrointestinal Bleeding OR Tumor OR Stenosis) AND (Argon Plasma Coagulation OR Argon Cautery OR Electrocautery OR Cautery OR Cauterization) | 19 |
| Cochrane CENTRAL | (Explosion OR Colonic explosion) AND (Colonoscopy OR Colon OR Adenoma OR Polyp OR Radiation Proctitis OR Gastrointestinal Bleeding OR Tumor OR Stenosis) AND (Argon Plasma Coagulation OR Argon Cautery OR Electrocautery OR Cautery OR Cauterization) | 1 |

**Supplemental Table 2.** Quality Assessment of the Included Prospective Cohort Studies using the NewCastle Ottawa Scale (NOS)

| **Study ID** | **Selection** | | | | | **Comparability** | **Outcomes** | | | | **Total Score (9**★**)** |
| --- | --- | --- | --- | --- | --- | --- | --- | --- | --- | --- | --- |
|  | **Representativeness of Exposed Cohort** (★**)** | **Selection of Non-exposed Cohort (**★**)** | **Ascertainment of Exposure (**★**)** | **Outcome of Interest not Present at the Start (**★**)** | **(**★★**)** | | **Assessment of Outcome (**★**)** | **Was the Follow-up Long Enough (**★**)** | **Adequacy of Follow-up (**★**)** |  | |
| Karamanolis, 2009 | ★ | ★ | ★ | ★ | ★★ | | ★ | ★ | ★ | ★★★★★★★★★ (9/9) | |

**Supplemental Table 3.** Results of Joanna Briggs Institute (JBI) Critical Appraisal Tool for the Quality Assessment of the Included Case Series

| **Study** | **1** | **2** | **3** | **4** | **5** | **6** | **7** | **8** | **9** | **10** | **Total Score (/10)** |
| --- | --- | --- | --- | --- | --- | --- | --- | --- | --- | --- | --- |
| Ben-Sousann, 2004 | Yes | Yes | Yes | Unclear | Unclear | Yes | Yes | Yes | Unclear | Yes | 7/10 |

**Questions:**
1) Were there clear criteria for the inclusion of case series?
2) Was the condition measured in a standard, reliable way for all participants included in the case series?
3) Were valid methods used for the identification of the condition for all participants included in the case series?
4) Did the case series have consecutive inclusion of participants?
5) Did the case series have a complete inclusion of participants?
6) Was there clear reporting of the demographics of the participants in the study?
7) Was there clear reporting of clinical information of the participants?
8) Were the outcomes or follow-up results of cases clearly reported?
9) Was there clear reporting of the presenting sites’/clinics’ demographic information?
10) Was statistical analysis appropriate?
